# Factors Associated With Mortality Among the COVID-19 Patients Treated at Gulu Regional Referral Hospital: A Retrospective Study

**DOI:** 10.3389/fpubh.2022.841906

**Published:** 2022-04-11

**Authors:** Steven Baguma, Christopher Okot, Nelson Onira Alema, Paska Apiyo, Paska Layet, Denis Acullu, Johnson Nyeko Oloya, Denis Ochula, Pamela Atim, Patrick Odong Olwedo, Smart Godfrey Okot, Freddy Wathum Drinkwater Oyat, Janet Oola, Eric Nzirakaindi Ikoona, Judith Aloyo, David Lagoro Kitara

**Affiliations:** ^1^Uganda Medical Association (UMA), UMA-Acholi Branch, Gulu, Uganda; ^2^Gulu Regional Referral Hospital, Gulu, Uganda; ^3^Department of Anatomy, Faculty of Medicine, Gulu University, Gulu, Uganda; ^4^Department of Medicine, St. Mary's Hospital, Lacor, Gulu, Uganda; ^5^Aga Kan Hospital, Mombasa, Kenya; ^6^Lamwo District Local Government, District Health Office, Padibe, Uganda; ^7^St. Joseph's Hospital, Kitgum, Uganda; ^8^Amuru District Local Government, District Health Office, Amuru, Uganda; ^9^Ambrosoli Hospital, Kalongo, Uganda; ^10^Nwoya District Local Government, District Health Office, Anaka, Uganda; ^11^ICAP at Columbia University, Freetown, Sierra Leone; ^12^Rhites-N, Acholi, Gulu, Uganda; ^13^Department of Surgery, Faculty of Medicine, Gulu University, Gulu, Uganda

**Keywords:** COVID-19, Gulu Hospital, mortality, comorbidities, female

## Abstract

**Background:**

The advent of the novel coronavirus disease 2019 (COVID-19) has caused millions of deaths worldwide. As of December 2021, there is inadequate data on the outcome of hospitalized patients suffering from COVID-19 in Africa. This study aimed at identifying factors associated with hospital mortality in patients who suffered from COVID-19 at Gulu Regional Referral Hospital in Northern Uganda from March 2020 to October 2021.

**Methods:**

This was a single-center, retrospective cohort study in patients hospitalized with confirmed SARS-CoV-2 at Gulu Regional Referral Hospital in Northern Uganda. Socio-demographic characteristics, clinical presentations, co-morbidities, duration of hospital stay, and treatments were analyzed, and factors associated with the odds of mortality were determined.

**Results:**

Of the 664 patients treated, 661 (99.5%) were unvaccinated, 632 (95.2%) recovered and 32 (4.8%) died. Mortality was highest in diabetics 11 (34.4%), cardiovascular diseases 12 (37.5%), hypertensives 10 (31.3%), females 18 (56.3%), ≥50-year-olds 19 (59.4%), no formal education 14 (43.8%), peasant farmers 12 (37.5%) and those who presented with difficulty in breathing/shortness of breath and chest pain 32 (100.0%), oxygen saturation (SpO_2_) at admission <80 4 (12.5%), general body aches and pains 31 (96.9%), tiredness 30 (93.8%) and loss of speech and movements 11 (34.4%). The independent factors associated with mortality among the COVID-19 patients were females AOR = 0.220, 95%CI: 0.059–0.827; *p* = 0.030; Diabetes mellitus AOR = 9.014, 95%CI: 1.726–47.067; *p* = 0.010; Ages of 50 years and above AOR = 2.725, 95%CI: 1.187–6.258; *p* = 0.018; tiredness AOR = 0.059, 95%CI: 0.009–0.371; *p* < 0.001; general body aches and pains AOR = 0.066, 95%CI: 0.007–0.605; *p* = 0.020; loss of speech and movement AOR = 0.134, 95%CI: 0.270–0.660; *p* = 0.010 and other co-morbidities AOR = 6.860, 95%CI: 1.309–35.957; *p* = 0.020.

**Conclusion:**

The overall Gulu Regional Hospital mortality was 32/664 (4.8%). Older age, people with diabetics, females, other comorbidities, severe forms of the disease, and those admitted to HDU were significant risk factors associated with hospital mortality. More efforts should be made to provide “*additional social protection*” to the most vulnerable population to avoid preventable morbidity and mortality of COVID-19 in Northern Uganda.

## Introduction

Coronavirus disease 2019 (COVID-19) caused by severe acute respiratory syndrome coronavirus 2 (SARS-CoV-2) first emerged in Hubei Province, China in December 2019 (1). Since then, not only has COVID-19 been considered a public health emergency of international concern, but it has been declared a global pandemic (1). COVID-19 is an infectious disease caused by a novel coronavirus that can be transmitted from one infected person to an average of three other people in a population (2). This emerging disease may be epidemiologically similar to severe acute respiratory syndrome (SARS) and the Middle East Respiratory Syndrome (MERS) in the context of its association with environmental and animal factors (3). Authors have suggested individual behavior will be crucial in controlling the spread of COVID-19 where early self-isolation, seeking medical advice remotely unless symptoms were severe, and social distancing was vital (4). Although most infected persons develop mild symptoms, some may develop respiratory failure, arrhythmia, shock, renal failure, cardiovascular injury, hepatic failure, and sometimes death (5). It is now reported that most symptoms occur because of inflammation (5).

At present, there is no approved antiviral treatment; only supportive care may be useful, for example, mechanical ventilation, extracorporeal membrane oxygenation (ECMO) to patients with refractory hypoxemia, or ECMO to patients with refractory hypoxemia (5). The overall Case Fatality Rate (CFR) for COVID-19 worldwide is 3.8% (5). CFR in patients with cardiovascular diseases, diabetes, hypertension, respiratory diseases, and cancers is estimated to be 13.2, 9.2, 8.4, 8.0, and 7.6%, respectively (6). Several studies focusing on factors affecting the mortality of COVID-19 have been published in medical journals (4–6).

Even though most African countries have fragile health systems, the Case Fatality Rate (CFR) for COVID-19 in Africa is surprisingly lower than the global trend (7). Lower positive test rates, a younger population, humid temperatures, and a possibility of a pre-existing immunity are some of the postulated factors associated with this difference since only the most severe cases of COVID-19 get tested and confirmed (7).

Although the SARS-CoV-2 virus predominantly targets the respiratory system, its associated mortality involves multiple organ systems (8). An increasing understanding of the disease throughout the pandemic has reduced hospital mortality rates, especially in well-resourced and high-income countries (9–11). In contrast, hospital mortality remains comparatively high in Africa (12). This has been attributed to the burden of underlying co-morbidities and resource deficits (12). Reports globally have indicated that increasing age (13–15), co-morbidities (cardiovascular diseases and diabetes) (13, 16, 17), and obesity (18, 19) are associated with adverse outcomes. In addition, certain demographic characteristics (14–16, 20) and laboratory parameters (21–23) have as well been associated with the severe form of COVID-19 and increased mortality.

The objective of this study was to identify factors associated with hospital mortality in patients who suffered from COVID-19 at Gulu Regional Referral Hospital in Northern Uganda from March 2020 to October 2021.

## Materials and Methods

### Study Design

This was a retrospective cohort study. Data review and abstraction of all COVID-19 hospital admissions registered in the Gulu Regional Referral Hospital Health Management Information System (HMIS) database and other tools were conducted. The period of the review was from March 2020 to October 2021(20 months). HMIS is a database established by the Ugandan Ministry of Health as a primary source of information on COVID-19 hospital admissions and deaths. COVID-19 notification is compulsory in Uganda, and the emergency operations Center at the Uganda National Public Health Institute receives reports on patients admitted to both public and private hospitals with COVID-19.

### Study Site

This study was conducted at Gulu Regional Referral Hospital in Northern Uganda, covering admissions of COVID-19 patients from March 2020 to October 2021. Gulu Hospital is a regional and referral center for patients from mid-northern Uganda. However, it receives patients from neighboring countries, for example, South Sudan and the Democratic Republic of Congo (DR Congo). It is a teaching hospital for Gulu University Medical School and many other health training institutions in the region. It is a 394-bed capacity hospital with outpatient and inpatient services estimated at 120,000 patients per year. The hospital has specialized units for example internal medicine, surgery, pediatrics, reproductive health, TB, HIV, cardiac, chest, dental, dermatology, sickle cell disease, diabetes, hypertension, ear, nose and throat, nutrition, accident, and emergency, laboratory, ophthalmology, mental health, and orthopedic clinics that are managed by consultants from Gulu Regional Referral Hospital and Gulu University.

Gulu Regional Referral Hospital was designated by the Ugandan Ministry of Health as a treatment center for COVID-19 patients in March 2020 when COVID-19 was declared a pandemic. A particular treatment unit for the management of COVID-19 (Gulu CTU) was established with a fully-fledged high dependency unit (HDU), with Oxygen supply and staff to manage the center. The COVID-19 isolation unit is housed in a separate building from other patients and comprises the general isolation ward and the COVID-19 Critical Care Unit (CCU). The CCU is split into a quasi-intensive care unit (ICU) and the high dependency unit (HDU). The general isolation ward consisted of two separate wards (one for females and another for males) with 12 beds. The COVID-19 CCU has a two-bed ICU and four-bed HDU. A multidisciplinary team of physicians, medical officers, nurses, and laboratory technicians managed the COVID-19 patients. The HDU and quasi-ICU were managed by an interdisciplinary team, including a full-time critical care specialist, primary physician, medical officers, physiotherapist, mental health specialists, and dietician. However, the ICU could not provide invasive procedures such as invasive mechanical ventilation, invasive hemodynamic monitoring, and inotropic support. All COVID-19 patients that required invasive procedures were transferred to Mulago National Referral Hospital for further management. The HDU served as a step-down unit for the quasi-ICU and housed critically ill patients requiring high-flow oxygen. Patients that required hemodialysis were transferred to Mulago National Referral Hospital for treatment in a separate and designated dialysis unit. The ICU and HDU had round-the-clock coverage with a physician a team of medical and clinical officers from various specialties such as internal medicine and emergency medicine. The nurse-to-patient ratio for ICU and HDU was 1:1 and 1:5, respectively. The team leader for the Gulu CTU was a consultant physician who managed all the COVID-19 patients admitted to the unit. In addition, support for the management of the COVID-19 patients at the Center was provided by the Ugandan Ministry of Health and World Health Organization experts using standard protocols developed and practiced in Uganda.

### Sources of Data

For the period of this study, patients admitted to Gulu Regional Referral Hospital with COVID-19 were estimated at 950. For each patient registered in the Gulu Hospital HMIS database, information on individual's socio-demographic characteristics, self-reported symptoms, signs, co-morbidities, COVID-19 Treatment Unit (CTU) admissions, HDU admissions, ICU admissions, and ventilatory support, dates of symptom onset, date of hospital admission, date of discharge, duration of hospital stay, reported circumstances when the disease was contracted, vaccination status and hospital outcomes (deaths, referrals, and releases/discharges) were included.

HMIS datasets were accessed, which were already de-identified and publicly available documents. Following ethically agreed principles on open data access, this review did not require stringent ethical approval in Uganda as we mainly worked on medical records with no identifiers included. However, we obtained ethical and administrative clearance from the Gulu Regional Referral Hospital Institution and Ethical Review Committee to access the archived Gulu Regional Referral Hospital datasets on COVID-19 patients.

### Selection Criteria

#### Inclusion Criteria

The following were the inclusion criteria for participants (i) Confirmed cases of SARS-CoV-2 with positive RT-PCR results (ii) patients 12 years and above (iii) completed information on the admission chart and other medical tools (iv) admission files and records on the HMIS.

#### Exclusion Criteria

We excluded (i) Incomplete medical records, (ii) records with no positive RT-PCR results on files (iii) participants below 12 years.

### Selection of Records

The medical records for the COVID-19 patients in the archives of Gulu Hospital were accessed. The selection of the COVID-19 patients' files was conducted consecutively and reviewed by the research team. The selection criteria were applied to each admission file (a total of nine hundred and forty-four files) (944); seven files (7) were excluded due to lack of RT-PCR results on files; thirteen (13) patients were less than twelve years; fifty-six (56) patients had incomplete files; ninety-six (96) patients appeared in HMIS database without admission files; one hundred and eight (108) patients had insufficient medical history on the file, and finally after excluding these files, a total of six hundred and sixty-four (664) files were included from the participating medical records for this research.

### Sample Size

The sample size for the study population was determined after applying the selection criteria on the medical records in the Gulu Hospital HMIS records. Six hundred and sixty-four (664) records were included as the sampled population.

### Training of Research Assistants

To obtain good and clean information from the COVID-19 patients' files, the research team trained the research assistants who were four in number (Two medical officers, one clinical officer, and one nurse) on how to use the selection criteria, accurately record data from the admission forms and exclude forms that were considered incomplete. The research teams were trained on infection, prevention, and control of the COVID-19 and were required to use facemasks, eye shields, and hand sanitizers during and after reviewing documents. The corresponding author supervised the data collection exercise from the beginning to the end, ensuring that every file was checked to confirm the completeness of the data collected.

### Data Collection Procedures

Registered COVID-19 patients treated at the Gulu Regional Referral Hospital with a positive quantitative RT-PCR test result for SARS-CoV-2 admitted to Gulu Hospital were consecutively reviewed. SARS-CoV-2 diagnostic tests followed national and international standards. They were done in certified laboratories of Gulu Regional Referral Hospital and Uganda Virus Research Institute (UVRI) as required by the Ugandan Ministry of Health and World Health Organization (WHO) protocols.

### Variables for the Study

The dependent variable for this study was the treatment outcomes (alive or dead). The independent variables were the socio-demographics of the COVID-19 patients (age, sex, occupation, religion, tribe, districts, and level of education), co-morbidities and treatments used, oxygen saturation at admission, dates of discharge from the hospital, duration of hospital stay, disease severity, and others), clinical presentations (signs and symptoms), vaccination status, residences, and circumstances under which the patient contracted the virus. Note: it typically took Gulu Regional Referral Hospital 24–48 h to obtain test positive confirmation results of SARS-CoV-2 from the Uganda Virus Research Institute (UVRI). For the asymptomatic and symptomatic cases, the time lag between diagnosis and admission was usually between 24 and 48 h for those whose residences and phone numbers were known by the COVID-19 district task forces.

At the beginning of the COVID-19 pandemic in March 2020 and due to extreme fear of the COVID-19, cases and contacts were reported very quickly to the Gulu Hospital (within 24 h) for fear of complications, the possibility of spreading the infection to their families, and death.

### Data Analysis

The analysis period was from the epidemiological week (starting month and date of March 2020) to the epidemiological week (until month and date of October 2021). The analysis was pre-specified and defined before reading the medical data in the Gulu Regional Referral Hospital records. The sample size was all patients (aged ≥12 years) with SARS-CoV-2 diagnosis who were admitted and registered to the Gulu Regional Referral Hospital HMIS database between the epidemiological weeks of March 2020 to October 2021. Means, frequencies, standard deviations, histograms, and percentages were used to summarize continuous variables, while frequencies and proportions were calculated for categorical variables. Age-adjusted and sex-adjusted rates for each district were calculated by the direct method using the estimated Ugandan population for 2020 as reference. We used the Chi-Square tests to observe associations between independent and dependent variables at 95% Confidence Intervals. Factors with *p*-values less or equal to 0.2 were entered into a multivariable logistical regression analysis to determine factors associated with mortality among COVID-19 patients treated at Gulu Regional Referral Hospital. However, the Gulu Regional Referral Hospital HMIS database contained many missing variables, for example, the reported symptoms, drugs used, and co-morbidities. We used additional Gulu Regional Referral Hospital records to fill in the missing data. Also, in the *post-hoc* analysis, we evaluated the missing data pattern and conducted a sensitivity analysis via multiple imputations by chained equations, generating 30 imputed datasets. All analyses were performed with SPSS version 25.0. Multiple imputations were performed with SPSS following the STROBE guideline recommendations. In addition, Adjusted Odds Ratios (AOR) for independent variables associated with mortality were calculated for the COVID-19 patients treated at the Gulu Regional Referral Hospital from March 2020 to October 2021.

### Ethical Considerations

This retrospective data review of COVID-19 patients' medical files at the Gulu Regional Referral Hospital was approved by the Gulu Hospital Institutional, Ethics, and Review Committee.

## Results

The study showed that during the period of study from March 2020, when the COVID-19 was declared a pandemic and Gulu Regional Referral Hospital became a treatment center, 32/664 (4.8%) COVID-19 patients died. Most patients who died were females AOR = 0.220, 95%CI: 0.059–0.827; *p* = 0.030; had Diabetes mellitus AOR = 9.014, 95%CI: 1.726–47.067; *p* = 0.010; and with co-morbidities for example cardiovascular diseases, other co-morbidities (hepatitis B, liver failure and HIV and AIDS) AOR = 6.860, 95%CI: 1.309–35.957; *p* = 0.020 and ages 50 years and above AOR = 2.725, 95%CI: 1.187–6.258; *p* = 0.018. Similarly, COVID-19 patients who presented with clinical symptoms for example tiredness AOR = 0.059, 95%CI: 0.009–0.371; *p* < 0.001; general body aches and pains AOR = 0.066, 95%CI: 0.007–0.605; *p* = 0.020, and loss of speech and movement AOR = 0.134, 95%CI: 0.270–0.660; *p* = 0.010 died. Nevertheless, most COVID-19 patients treated at the Gulu Regional Referral Hospital were unvaccinated 661/664 (99.5%) against the coronavirus and the recovery rate from the disease was 632/664 (95.2%).

Table 1 shows that most COVID-19 mortality at Gulu Regional Referral Hospital from March 2020 to October 2021 were females 18 (56.3%), age group >50 years 19 (59.4%); no formal education 14 (43.0%), Acholi 25 (78.1%), Catholics 13 (40.6%), Peasant farmers 12 (37.5%) and from Gulu District 15 (46.9%).

**Table 1:** Socio-demographic characteristics of the COVID-19 mortality at Gulu Regional Referral Hospital.

**Variables**	**Frequency**	**Percent (%)**
**Gender**		
Male	14	43.7
Female	18	56.3
**Age (years)**		
<20	0	0.0
20–29	3	9.4
30–39	1	3.1
40–49	9	28.1
>50	19	59.4
**Tribes**		
Acholi	25	78.1
Lango	1	3.1
Baganda	0	3.1
Madi	1	3.1
Others	4	12.5
**Religion**		
Catholics	13	40.6
Protestants	5	15.6
Born Again	1	3.1
Muslims	3	9.4
Others	10	31.3
**The highest level of education attained**		
No formal education	14	43.0
Primary	0	0.0
Secondary	0	0.0
Certificates	5	15.6
Diploma	3	9.4
Degrees	5	15.6
Postgraduate degrees	2	6.3
**Occupation**		
Business	2	6.3
Civil Servants	2	6.3
Health workers	3	9.4
Teachers	1	3.1
Uniformed security forces	0	0.0
Peasant Farmers	12	37.5
Others	12	37.5
**Districts**		
Agago	1	3.1
Amuru	2	6.3
Gulu	15	46.9
Kitgum	1	3.1
Lamwo	0	0.0
Nwoya	2	6.3
Omoro	6	18.8
Pader	1	3.1
Others	4	12.5
Number of COVID-19 patients who died	32	4.8

In Table 2, factors associated with mortality at bivariate analysis were cough χ^2^ = 10.639; *p* = 0.000; tiredness χ^2^ = 6.488; *p* = 0.000; Age (>50 years) χ^2^ = 40.601; *p* =0.000; no formal education χ^2^ = 39.213; *p* = 0.000; peasant farmers χ^2^ = 119.828; *p* = 0.000; general body aches and pains χ^2^ = 75.543; *p* = 0.000; diarrhea χ^2^ = 10.336; *p* = 0.001; difficulty in breathing/shortness of breath/chest pain χ^2^ = 96.929; *p* = 0.000; loss of speech and movement χ^2^ = 113.202; *p* = 0.001; headache χ^2^ = 9.705; *p* = 0.002; diabetes mellitus χ^2^ = 51.156; *p* = 0.000; Other CVDs χ^2^ = 34.819; *p* = 0.000; hypertension χ^2^ = 10.807; *p* = 0.000; HIV and AIDS χ^2^ = 6.488; *p* = 0.011; oxygen saturation at admission (SpO_2_) < 80) χ^2^ = 62.074; *p* = 0.000; duration of hospital stay (0–1 week) 33.235; *p* = 0.000; Catholics χ^2^ = 28.691; *p* = 0.000; Gulu district χ^2^ = 21.827; *p* = 0.040 and females χ^2^ = 7.986; *p* = 0.005.

**Table 2:** Factors associated with mortality at the bivariate analysis on the COVID-19 patients treated at Gulu Regional Referral Hospital from March 2020 to October 2021.

**Variables**	**Freq *n* = 32 (%)**	**Chi-square**	**df**	***p*-value**
Fever	9 (28.1)	3.143	1	0.076
Cough	25 (78.1)	10.639	1	0.000
Tiredness	30 (93.8)	119.828	1	0.000
General body aches and pains	31 (96.9)	75.543	1	0.000
Diarrhea	4 (12.5)	10.336	1	0.001
Difficulty in breathing/shortness of breath/chest pain	32 (100.0)	96.929	1	0.000
Loss of speech and movement	11 (34.4)	113.202	1	0.000
Headache	19 (59.4)	9.705	1	0.002
Sore throat	8 (25.0)	1.251	1	0.263
Rashes on the skin and discoloration of toes and fingers	1 (3.1)	3.579	1	0.059
Loss of smell	0 (0.0)	2.328	1	0.127
Loss of taste	1 (3.1)	0.669	1	0.413
Diabetes mellitus	11 (34.4)	51.156	1	0.000
Chronic Obstructive Pulmonary Diseases (COPD)	1 (3.1)	1.041	1	0.307
Other cardiovascular diseases (CVDs)	12 (37.5)	34.819	1	0.000
Hypertension	10 (31.3)	10.807	1	0.001
Obesity	0 (0)	0.153	1	0.696
Asthma	1 (3.1)	0.113	1	0.737
Cancer	1 (3.1)	3.565	1	0.059
HIV and AIDS	7 (21.9)	6.488	1	0.011
Symptomatic	28 (87.5)	2.170	1	0.141
Oxygen saturation at admission (SpO_2_)	(<80) 4 (12.5)	62.074	2	0.000
Duration of hospital stay (0–1 week)	25 (78.1)	67.776	1	0.000
Duration of symptoms (1–7 days)	19 (59.4)	1.101	3	0.897
**Systolic blood pressure (mmHg)**				
<120 mmHg	13 (40.6)			
121–140 mmHg	12 (37.5)	2.920	2	0.232
>140 mmHg	7 (21.9)			
**Diastolic blood pressure (mmHg)**				
<80 mmHg	22 (68.8)			
81–120 mmHg	4 (12.5)	4.214	2	0.122
121–140	6 (18.8)			
**Duration of symptoms (days)**				
1–7	19 (59.4)			
8–14	10 (31.3)	1.101	4	0.894
15–21	3 (9.4)			
22–28	0 (0.0)			
**Ages of participants (years)**				
<20	0 (0.0)			
20–29	3 (9.4)			
30–39	1 (3.1)			
40–49	9 (28.1)			
>50	19 (59.4)	40.601	4	0.000
**The highest level of education attained**				
No formal education	14 (43.8)	39.213	8	0.000
Primary level	0 (0.0)			
Secondary level	0 (0.0)			
Certificates	5 (15.6)			
Diploma	3 (9.4)			
Degrees	5 (15.6)			
Postgraduate degree	2 (6.3)			
**Tribes**				
Acholi	25 (78.1)	9.511	7	0.218
Langi	0 (0.0)			
Baganda	1 (3.1)			
Lugbara	1 (3.1)			
Others	4 (12.5)			
**Occupation**				
Peasant farmers	12 (37.5)	33.235	8	0.000
Business	2 (6.3)			
Uniformed security	0 (0.0)			
Civil servants	2 (6.3)			
Teachers	1 (3.1)			
Health workers	3 (9.4)			
Others	12 (37.5)			
**Religion**				
Born again	1 (3.1)			
Catholic	13 (40.6)	28.691	5	0.000
Moslems	3 (9.4)			
Protestants	5 (15.6)			
Others				
**Districts**			
Agago	1 (3.1)			
Amuru	2 (6.3)			
Gulu	15 (46.9)	21.827	12	0.040
Kitgum	1 (3.1)			
Lamwo	0 (0.0)			
Nwoya	2 (6.3)			
Omoro	6 (18.8)			
Pader	1 (3.1)			
Others	4 (12.5)	107.107	69	0.002
**Gender**				
Females	18 (56.3)	7.986	1	0.005
Males	14 (43.7)			

*COPD, Chronic obstructive pulmonary diseases; CVDs, cardiovascular diseases*.

In Table 3, the Adjusted Odds Ratios (AOR) of factors associated with mortality among COVID-19 patients treated at Gulu Regional Referral Hospital were; Females AOR = 0.220, 95%CI: 0.059–0.827; *p* = 0.030; Diabetes mellitus AOR = 9.014, 95%CI: 1.726–47.067; *p* = 0.010; Tiredness AOR = 0.059, 95%CI: 0.009–0.371; *p* < 0.001; general body aches and pains AOR = 0.066, 95%CI: 0.007–0.605; *p* = 0.020; loss of speech and movement AOR = 0.134, 95%CI: 0.270–0.660; *p* = 0.010; ages 50 years and above AOR = 2.725, 95%CI: 1.187–6.258; *p* = 0.018 and other co-morbidities AOR = 6.860, 95%CI: 1.309–35.957; *p* = 0.020.

**Table 3:** The adjusted Odds Ratios for factors associated with mortality among COVID-19 patients treated at Gulu Regional Referral Hospital.

**Variables**	**AOR**	**95% CI**	***p*-value**
Females	0.220	0.059–0.827	0.030
Diabetes mellitus	9.014	1.726–47.067	0.010
Ages 50 years and above	2.725	1.187–6.258	0.018
Other co-morbidities	6.860	1.309–35.957	0.020
Cancers	1.461	0.022–99.174	0.860
HIV and AIDs	3.041	0.653–14.166	0.157
Certificate of education	4.698	0.596–37.023	0.142
Other cardiovascular diseases (CVDs)	7.050	0.179–277.547	0.297
Chronic obstructive pulmonary diseases (COPDS)	0.441	0.016–12.106	0.628
Tiredness	0.059	0.009–0.371	0.000
General body aches and pains	0.066	0.007–0.605	0.020
Loss of speech and movements	0.134	0.270–0.660	0.010
Vomiting	1.209	0.149–9.804	0.859
Diarrhea	3.167	0.360–27.853	0.299
Sore throat	0.478	0.110–2.073	0.324

*Other co-morbidities include hepatitis B, liver failure, and liver cirrhosis*.

In Table 4, the female COVID-19 patients were statistically and significantly associated with cardiovascular diseases (χ^2^ = 4.996; *p* = 0.025) and Chronic Obstructive Pulmonary Diseases (COPDs) χ^2^ = 6.346; *p* = 0.032, and with close to significant associations with HIV and AIDS (χ^2^ = 3.646; *p* = 0.056) and Cancers χ^2^ = 3.144; *p* = 0.076).

**Table 4:** Crosstabulations on factors associated with female gender among the COVID-19 patients in Gulu Hospital.

**Variables**	**Chi-square**	**df**	***p*-value**
**Crosstabulations between duration of symptoms (days) and other variables**			
Symptomatic patients	10.301	4	**0.036**
Age of patients	14.585	16	0.555
Female patients	6.284	4	0.179
The highest level of education attained	30.42	32	0.547
Crosstabulations between Diabetes Mellitus and other variables
Symptomatic patients	5.314	1	**0.021**
Age of patients	22.66	4	**0.000**
Female patients	0.016	1	0.901
The highest level of education attained	32.532	8	**0.000**
Crosstabulations between Chronic obstructive pulmonary diseases (COPDs) and other variables			
Symptomatic patients	0.014	1	0.905
Age of patients	6.195	4	0.185
Female patients	6.346	1	**0.032**
The highest level of education attained			
Crosstabulations between other cardiovascular diseases (CVDs) and other variables			
Symptomatic patients	4.462	1	**0.035**
Age of the patients	22.562	1	**0.000**
Female patients	4.996	1	**0.025**
The highest level of education attained	22.451	8	**0.004**
Crosstabulations between hypertension and other variables			
Symptomatic patients	3.045	1	0.081
Age of the patients	35.169	4	**0.000**
Female patients	1.187	1	0.276
The highest level of education attained	21.624	8	**0.006**
Crosstabulations between obesity and other variables			
Symptomatic patients	0.173	1	0.678
Age of patients	1.272	4	0.866
Female patients	1.512	1	0.219
The highest level of education attained	2.083	8	0.978
Crosstabulations between Asthma and other variables			
Symptomatic patients	0.0920	1	0.762
Age of patients	3.7700	4	0.438
Female patients	2.8110	1	0.094
The highest level of education attained	10.3790	8	0.239
Crosstabulations between Cancer and other variables			
Symptomatic patients	1.2200	1	0.269
Age of patients	1.7470	4	0.782
Female patients	3.1440	1	0.076
The highest level of education attained	12.6480	8	0.125
Crosstabulations between HIV and AIDS and other variables			
Symptomatic patients	0.070	1	0.791
Age of patients	8.515	4	0.074
Female patients	3.646	1	0.056
The highest level of education attained	2.107	8	0.978

*The bold values are those that were statistically significant*.

## Discussions

The most significant finding from this study was the low mortality rate among COVID-19 patients treated at Gulu Regional Referral Hospital 32/664 (4.8%) (Table 1). This mortality rate was much lower than the average mortality rates reported in Africa (6, 12) but slightly higher than the global average amongst hospitalized patients at 3.8% (6, 24). It may not be apparent to readers to find that several asymptomatic SARS-CoV-2 patients were admitted to the Gulu Regional Referral Hospital COVID-19 treatment Center. It is important to note that at the beginning of the COVID-19 pandemic in March 2020, the Ugandan government policy was that all COVID-19 cases and contacts were mandatorily isolated or admitted to the treatment Center until the SARS-CoV-2 test results became negative. This admission policy which was applied throughout the country on all SARS-CoV-2 test positive was maintained until March 2021, when the government adopted a home-based care approach for managing mild and contact cases of the COVID-19 from home.

We authors suggest the low mortality rates were attributable to the mild nature of the COVID-19 disease and relatively fewer patients admitted to the Gulu Regional Referral Hospital with co-morbidities. This was shown by the clinical presentations in most patients who had oxygen saturation (SpO_2_), more than ninety-six (96) at admission, and one-fourth of the patients were asymptomatic (Table 2). Furthermore, the admission of asymptomatic cases may appear strange but at the beginning of the COVID-19 in March 2020 and because of many uncertainties, the Government of Uganda adopted a policy of isolating all cases and contacts to the treatment or general holding centers to reduce contacts of the COVID-19 cases with the general population. This government policy provided additional social protection to the general Ugandan population from cases and contacts of the COVID-19 cases. This may have, in many ways, accounted for the non-exponential spread of the virus in Uganda during the pandemic in 2020 and early 2021.

At the same time, most symptomatic cases were mild, and most presented early to the hospital in the course of the disease (Table 2). In addition, the medical team of the Gulu Regional Referral Hospital CTU must be commended for the remarkable work done over the period in successfully treating over 900 COVID-19 patients in one year and eight months with very low mortality considering the resource scarcity. This could not have been achieved without the dedication and technical expertise of the medical team at the Gulu Regional Referral Hospital CTU. The study, however, identified several risk factors associated with mortality among hospitalized COVID-19 patients in the Ugandan setting. Our study found females, Diabetes mellitus, co-morbidities [cardiovascular diseases, hepatitis B, HIV and AIDS, Chronic Obstructive pulmonary diseases (COPDs), and liver failure], and severe illnesses as shown by symptoms such as tiredness, general body aches, and pains, and loss of speech and movements as the risk factors associated with higher odds of mortality (Table 3). This finding was similarly observed in other studies (6, 13, 16, 17) where co-morbidities and severe symptoms and signs of COVID-19 were associated with mortality. Symptoms such as tiredness, general body aches, and pains, loss of speech and movements were observed in most COVID-19 patients who developed the severe disease and died.

Furthermore, this study found a higher chance of death in COVID-19 patients with Diabetes mellitus (Table 3). Authors have stressed the importance of glycemic control during COVID-19 infections because hyperglycemia may adversely affect pulmonary functions and immune response (25). Studies found that hyperglycemia secondary to Diabetes mellitus leads to immune dysfunction by impairing humoral and cellular functions and the antioxidant systems (26, 27). In addition, studies showed that diabetic patients were more susceptible to nosocomial infections (26, 27). These factors may have contributed to higher chances of death in diabetic patients with COVID-19 (28), which we also observed in our study population.

Also, the age group of 50 years and above 19 (59.4%) were the majority among those who died. It was statistically significant at bivariate analysis in the current study (Age >50 years) χ^2^ = 40.601; *p* = 0.000 (Table 2) and at the multivariable logistic regression analysis AOR = 2.725, 95%CI: 1.187–6.258; *p* = 0.018. This finding is consistent with previous studies that showed that the elderly were more likely to suffer from more severe forms of the disease (8, 29, 30). In many studies, the median age of hospitalized patients with a severe form of the COVID-19 ranged from 49 to 56 years (8, 29, 30). This was consistent with reports published globally and comparable to other African studies (12, 31).

Furthermore, we argue that the older age group (>50 years and above) in our study had similarly, a statistically significant association with mortality and increased odds of mortality (Table 3). We note that although all age groups are at risk of contracting COVID-19, older people face a significantly higher risk of developing severe illness if they contract the disease due to physiological changes associated with aging and potential underlying health conditions (32). In addition, there are very few studies that connect the known mechanisms of aging to the pathogenesis of viruses however, we have been persuaded with potential mechanistic explanations as to why COVID-19 advances in some people and not others, especially in older patients, including differences in the immune system, glycation, the epigenome, inflammasome activity, and biological age (32). We argue that the immune system changes in many ways during aging, including a gradual decline in the immune function called immunosenescence, which hampers pathogen recognition, alert signaling, and clearance (32). This is an aging-related phenomenon whereby old or dysfunctional cells arrest their cell cycle and become epigenetically locked into a pro-inflammatory state (32). The aging cells secrete cytokines and chemokines, which appear to promote the severity of the illness due to the COVID-19 (32). In addition, during aging, the other classic immune system change is a chronic increase in systemic inflammation called inflame-aging, which arises from an overactive, yet ineffective alert system that seems to function more (33). These two scenarios have persuaded authors to argue that these phenomena may give plausible explanations for the higher mortality rates of COVID-19 among the elderly population. Furthermore, many studies have confirmed that the elderly and males were at increased risks of mortality due to the diseases (8, 29, 30). This was similarly observed in our study, where the middle-aged persons (>50-year-olds) were the most affected (Table 3).

However, contrary to many studies cited above, most mortalities in our cohort were among females (Table 3). This finding in many ways contrasts with previous results in many countries since the onset of the COVID-19 pandemic in 2019. We have asked questions about this development and still asking more questions about why and how females in Northern Uganda suffered more mortalities from COVID-19 than males (Tables 1, 3). Yet, more males were admitted with the disease than females (Table 3). Also, we found in the statistical analysis that females in Northern Uganda had statistically significant associations with cardiovascular diseases (χ^2^ = 4.996; *p* = 0.025) and chronic obstructive pulmonary diseases (COPDs) χ^2^ = 6.346; *p* = 0.032, with close significant associations with HIV and AIDs (χ^2^ = 3.646; *p* = 0.056) and cancers χ^2^ = 3.144; *p* = 0.076) (Table 4). Cardiovascular diseases are co-morbidities that lead to severe illness, hospitalization, and death from the COVID-19 (13, 16, 17). Furthermore, we argue that the high prevalence of HIV and AIDs and cancers among females in Northern Uganda may have contributed to the higher mortality rates observed among the female COVID-19 patients in the current study population. Recent studies from Northern Uganda showed a higher prevalence of HIV and AIDS among females in Northern Uganda at 17.1% than males at 8.0% (34). Similarly, most cancer prevalence was higher in Northern Uganda than in the rest of the country, especially breast and cervical cancers which commonly affect females (35). We argue that these four factors (cardiovascular diseases, chronic obstructive pulmonary diseases (COPDs), HIV and AIDs, and cancers) may have singly or collectively contributed to the higher mortality risks of the COVD-19 among females in Northern Uganda. Some scholars and academicians may argue that the higher mortality rates among females seen in this cohort may have resulted from the admission of only severe cases of female COVID-19 patients compared to the males. We found out that the Gulu Regional Referral Hospital practiced unprejudiced admission guidelines, and all patients (males or females) were admitted based on the SARS-CoV-2 positive test results. This has been further confirmed by near equal numbers of the asymptomatic COVID-19 patients (males and females) admitted to the Gulu CTU (Table 2).

In addition, tiredness, cough, diarrhea, difficulty in breathing, shortness of breath, chest pain, headache, general body aches and pains, loss of speech and movement were associated with COVID-19 mortality at Gulu Regional Referral Hospital (Tables 2–4). This finding is supported by many studies that showed that the symptoms and signs were more frequent in SARS cases and cases of death (29–31, 36).

In another study, dyspnea was the main symptom of SARS-CoV-2, a severe COVID-19 disease with more chances of death (36). However, other reported symptoms, such as headache, general body aches, and pains, diarrhea, loss of speech and movements, tiredness, cough, difficulty in breathing, shortness of breath, and chest pain were associated with higher chances of death among this cohort (Tables 2–4). Though, loss of smell and taste, sore throat, rashes on the skin, discoloration of the toes and nails, and runny nose were not statistically associated with death in this current study population (Tables 2, 3). This agrees partly with Patrícia Rezende do Prado et al., who reported dyspnea as the factor associated with COVID-19 death, while cough, fever, and other symptoms were protective factors (36).

Taking these results, it can be concluded that this study analyzed the epidemiological characteristics and mortality risk factors in individuals diagnosed with COVID-19 due to SARS-CoV-2 at Gulu Regional Referral Hospital in Northern Uganda. Our view is that the high-risk groups need special attention, especially the elderly and those with co-morbidities as diabetes mellitus, cancers, HIV and AIDs, cardiovascular diseases, hepatitis B, liver failure, and females with conditions. We, authors suggest special attention to be accorded to COVID-19 patients with dyspnea, general body pains and aches, loss of speech and movement, tiredness, headache, and diarrhea as they seem to develop very severe forms of the disease with many adverse outcomes. In addition, some symptoms that we recorded were more frequent in mild cases of COVID-19 and we propose that they should be elucidated in future studies.

Furthermore, females had more mortality risks in this current study mainly in part due to more comorbidities (cardiovascular diseases, chronic obstructive pulmonary diseases (COPDs), HIV and AIDS, and cancers) (Table 4). We propose that special care be accorded to females who contract COVID-19 to screen out for co-morbidities in the early management phases so that avoidable morbidity and mortality can be made.

## Strengths and Limitations of this Study

This study was a retrospective review of datasets from the COVID-19 medical records at Gulu Regional Referral Hospital from March 2020 to October 2021. The study has limitations on how the Gulu Regional Referral Hospital handled records and record keeping. In addition, vital information, such as weight, height, and BMI of COVID-19 patients, was not recorded due to the emergency handling of the cases at the beginning of the pandemic in March 2020. The missing variables in the Hospital HMIS records got a few files excluded from the participating records for this study. In this, we suggest a need for a prospective or a longitudinal assessment of the COVID-19 cases in the future, ensuring that all data are measured and recorded accordingly.

This data is vital as it is one of the well-documented and completed data for over 664 cases of COVID-19 treated at a regional referral hospital in Uganda. Findings from this study show good clinical practices at the Gulu Regional Referral Hospital despite the logistical challenges faced during the pandemic.

## Generalizability of the Results

These findings should be cautiously interpreted and generalized to regional referral hospitals in low-resource settings such as Uganda.

## Conclusion

The overall Gulu Regional Referral Hospital COVID-19 mortality was 4.8% (32/664). Older age groups (>50 years old), diabetics, females, and those with co-morbidities, severe forms of the disease, and admitted to HDU were significant risk factors associated with hospital mortality. More efforts should be made to offer additional social protection to the most vulnerable population from the general population to avoid preventable morbidity and mortality resulting from COVID-19 in Northern Uganda. Further studies are necessary to understand why females had higher mortality even after adjustments, possibly implying admission selection bias or different accessibility of hospital care for the females which have been clarified by the hospital authorities as equal to everybody irrespective of gender.

## Authors' Notes

EI is a Technical Director at ICAP at the University of Columbia, Sierra Leone; JO is a medical officer and member of the Uganda Medical Association, UMA-Acholi branch, Gulu City, Uganda; CO is a Medical Officer Special Grade, Department of surgery at Gulu Regional Referral Hospital, Gulu City, Uganda; SB is a Medical Officer in the Department of Obstetrics and Gynecology at Gulu Regional Referral Hospital, Gulu City, Uganda; PAp is a Consultant Physician at Gulu Regional Referral Hospital, Gulu City, Uganda; NO is a Lecturer at Gulu University, Faculty of Medicine, Department of Anatomy, Gulu City, Uganda; FO is a senior physician, a public health specialist, and member of Uganda Medical Association, UMA-Acholi branch, Gulu City, Uganda; PL was a senior clinician and public health specialist at St. Mary's Hospital Lacor, Gulu City, Uganda; DA is a senior Consultant Radiologist at the Aga Khan Hospital, Mombasa, Kenya; DO is a public health specialist and District Health officer at Lamwo district local government, Lamwo, Uganda; PAt is a senior Obstetrician and Gynecologist and Medical Superintendent at St. Joseph's Hospital, Kitgum, Uganda; PO is a senior public health specialist and a District Health Office of Amuru district Local Government, Uganda; SO is a senior surgeon and a Medical Superintendent of Ambrosoli Hospital, Kalongo, Agago district local government, Uganda; JO was a senior public health specialist and a District Health Officer of Nwoya district Local Government, Nwoya, Uganda; JL is a Technical Director at the Rhites-N, Acholi, Gulu City, Uganda; DK is a Takemi fellow of Harvard University and a Professor at Gulu University, Faculty of Medicine, Department of Surgery, Gulu City, Uganda.

## Data Availability Statement

The datasets presented in this study can be found in online repositories. The names of the repository/repositories and accession number(s) can be found in the article/Supplementary Material.

## Ethics Statement

The studies involving human participants were reviewed and approved by Gulu Regional Referral Hospital Research and Ethics Committee. Written informed consent to participate in this study was provided by the participants' legal guardian/next of kin.

## Author Contributions

DK, EI, PL, JNO, JA, JO, and FO participated in designing the study. SB and DK were responsible for data abstraction supervision. EI and DK were responsible for data analysis and interpretation. SB, CO, NA, JO, PAp, PAt, PL, DA, JNO, DO, PO, SO, FD, JA, and DK for writing and revising the manuscript. All authors approved the manuscript.

## Funding

Most funding for this study was contributions of individual research members of the Uganda Medical Association (UMA) Acholi branch.

## Conflict of Interest

The authors declare that the research was conducted in the absence of any commercial or financial relationships that could be construed as a potential conflict of interest.

## Publisher's Note

All claims expressed in this article are solely those of the authors and do not necessarily represent those of their affiliated organizations, or those of the publisher, the editors and the reviewers. Any product that may be evaluated in this article, or claim that may be made by its manufacturer, is not guaranteed or endorsed by the publisher.
