# Supplementary material for: Factors Associated With Mortality Among the COVID-19 Patients Treated at Gulu Regional Referral Hospital: A Retrospective Study
Source: Front Public Health. 2022 Apr 11;10:841906. doi: 10.3389/fpubh.2022.841906 (PMC9035511; doi:10.3389/fpubh.2022.841906)
Supplement: Supplementary file 1 [file Table_1.DOCX]

**Logistic Regression**

| **Case Processing Summary** | | | |
| --- | --- | --- | --- |
| Unweighted Cases | | N | Percent |
| Selected Cases | Included in Analysis | 637 | 95.9 |
|  | Missing Cases | 27 | 4.1 |
|  | Total | 664 | 100.0 |
| Unselected Cases | | 0 | .0 |
| Total | | 664 | 100.0 |
| a. If weight is in effect, see the classification table for the total number of cases. | | | |

| **Dependent Variable Encoding** | |
| --- | --- |
| Original Value | Internal Value |
| Death | 0 |
| Alive | 1 |

| **Categorical Variables Codings** | | | | |
| --- | --- | --- | --- | --- |
|  | | Frequency | Parameter coding | |
|  |  |  | (1) | (2) |
| Diastolic Blood Pressure | Less than 80 | 325 | 1.000 | .000 |
|  | 121-140 | 180 | .000 | 1.000 |
|  | Above 90 | 132 | .000 | .000 |
| Systolic Blood Pressure | Less than 120 | 176 | 1.000 | .000 |
|  | 121-140 | 294 | .000 | 1.000 |
|  | Above 140 | 167 | .000 | .000 |
| Loss of speech or movement | Yes | 20 | 1.000 |  |
|  | No | 617 | .000 |  |
| Cough | Yes | 319 | 1.000 |  |
|  | No | 318 | .000 |  |
| Tiredness | Yes | 126 | 1.000 |  |
|  | No | 511 | .000 |  |
| Aches and pains | Yes | 186 | 1.000 |  |
|  | No | 451 | .000 |  |
| Sore throat | Yes | 115 | 1.000 |  |
|  | No | 522 | .000 |  |
| Vomiting | Yes | 21 | 1.000 |  |
|  | No | 616 | .000 |  |
| Diarrhea | Yes | 18 | 1.000 |  |
|  | No | 619 | .000 |  |
| Conjunctivitis | Yes | 1 | 1.000 |  |
|  | No | 636 | .000 |  |
| Difficulty in breathing or shortness of breath  chest pain or pressure | Yes | 164 | 1.000 |  |
|  | No | 473 | .000 |  |
| Rash on the skin or discoloration of fingers or toes | Yes | 2 | 1.000 |  |
|  | No | 635 | .000 |  |
| Loss of smell | Yes | 39 | 1.000 |  |
|  | No | 598 | .000 |  |
| Loss of taste | Yes | 41 | 1.000 |  |
|  | No | 596 | .000 |  |
| Headache | Yes | 221 | 1.000 |  |
|  | No | 416 | .000 |  |
| Fever | Yes | 107 | 1.000 |  |
|  | No | 530 | .000 |  |

**Block 0: Beginning Block**

| **Classification Table^,b^** | | | | | | | | | | | | |  |
| --- | --- | --- | --- | --- | --- | --- | --- | --- | --- | --- | --- | --- | --- |
|  | | Observed | | | | | Predicted | | | | | |  |
|  | |  |  |  |  |  | Outcome (1/Death) | | | | Percentage Correct | |  |
|  | |  |  |  |  |  | Death | | Alive | |  |  |  |
| Step 0 | | Outcome (1/Death) | | | Death | | 0 | | 32 | | .0 | |  |
|  |  |  |  |  | Alive | | 0 | | 605 | | 100.0 | |  |
|  |  | Overall Percentage | | | | |  | |  | | 95.0 | |  |
| a. Constant is included in the model. | | | | | | | | | | | | |  |
| b. The cut value is .500 | | | | | | | | | | | | |  |
| **Variables in the Equation** | | | | | | | | | | | | | |
|  | | | B | S.E. | | Wald | | df | | Sig. | | Exp(B) | |
| Step 0 | Constant | | 2.939 | .181 | | 262.610 | | 1 | | .000 | | 18.906 | |

| **Variables in the Equation** | | | | | |
| --- | --- | --- | --- | --- | --- |
|  | | | Score | df | Sig. |
| Step 0 | Variables | Fever(1) | 3.093 | 1 | .079 |
|  |  | Cough(1) | 10.601 | 1 | .001 |
|  |  | Tiredness(1) | 116.180 | 1 | .000 |
|  |  | Aches and pains(1) | 74.643 | 1 | .000 |
|  |  | Sore throat(1) | 1.099 | 1 | .294 |
|  |  | Vomiting(1) | 16.063 | 1 | .000 |
|  |  | Diarrhea (1) | 11.484 | 1 | .001 |
|  |  | Conjunctivitis(1) | .053 | 1 | .818 |
|  |  | Systolic Blood Pressure | 2.848 | 2 | .241 |
|  |  | Systolic Blood Pressure(1) | 2.846 | 1 | .092 |
|  |  | Systolic Blood Pressure(2) | 1.015 | 1 | .314 |
|  |  | Diastolic Blood Pressure | 4.253 | 2 | .119 |
|  |  | Diastolic Blood Pressure(1) | 4.238 | 1 | .040 |
|  |  | Diastolic Blood Pressure(2) | 1.502 | 1 | .220 |
|  |  | Headache (1) | 9.059 | 1 | .003 |
|  |  | Loss of taste (1) | .613 | 1 | .433 |
|  |  | Loss of smell(1) | 2.197 | 1 | .138 |
|  |  | Rash on the skin or discoloration of fingers or toes(1) | 8.506 | 1 | .004 |
|  |  | Difficulty in breathing or shortness of breath  chest pain or pressure  (1) | 97.174 | 1 | .000 |
|  |  | Loss of speech or movement (1) | 108.091 | 1 | .000 |
|  | Overall Statistics | | 216.000 | 18 | .000 |

**Block 1: Method = Enter**

| **Omnibus Tests of Model Coefficients** | | | | | | | | | | | | |
| --- | --- | --- | --- | --- | --- | --- | --- | --- | --- | --- | --- | --- |
|  | | | | Chi-square | | | df | | | Sig. | | |
| Step 1 | | Step | | 168.316 | | | 18 | | | .000 | | |
|  |  | Block | | 168.316 | | | 18 | | | .000 | | |
|  |  | Model | | 168.316 | | | 18 | | | .000 | | |
| **Model Summary** | | | | | | | | | | |  |  |
| Step | -2 Log-likelihood | | | | Cox & Snell R Square | | | Nagelkerke R Square | | |  |  |
| 1 | 85.475^a^ | | | | .232 | | | .707 | | |  |  |
| a. Estimation terminated at iteration number 20 because maximum iterations have been reached. The final solution cannot be found. | | | | | | | | | | |  |  |
| **Hosmer and Lemeshow Test** | | | | | | | | | | | |  |
| Step | | | Chi-square | | | df | | | Sig. | | |  |
| 1 | | | .342 | | | 8 | | | 1.000 | | |  |

| **Contingency Table for Hosmer and Lemeshow Test** | | | | | | |
| --- | --- | --- | --- | --- | --- | --- |
|  | | Outcome (1/Death) = Death | | Outcome (1/Death) = Alive | | Total |
|  |  | Observed | Expected | Observed | Expected |  |
| Step 1 | 1 | 31 | 30.296 | 33 | 33.704 | 64 |
|  | 2 | 1 | 1.667 | 63 | 62.333 | 64 |
|  | 3 | 0 | .037 | 63 | 62.963 | 63 |
|  | 4 | 0 | .000 | 64 | 64.000 | 64 |
|  | 5 | 0 | .000 | 69 | 69.000 | 69 |
|  | 6 | 0 | .000 | 77 | 77.000 | 77 |
|  | 7 | 0 | .000 | 62 | 62.000 | 62 |
|  | 8 | 0 | .000 | 72 | 72.000 | 72 |
|  | 9 | 0 | .000 | 65 | 65.000 | 65 |
|  | 10 | 0 | .000 | 37 | 37.000 | 37 |

| **Classification Tables** | | | | | | | | | | | | | | |  |
| --- | --- | --- | --- | --- | --- | --- | --- | --- | --- | --- | --- | --- | --- | --- | --- |
|  | | Observed | | | | | | Predicted | | | | | | |  |
|  | |  |  |  |  |  |  | Outcome (1/Death) | | | | | Percentage Correct | |  |
|  | |  |  |  |  |  |  | Death | | Alive | | |  |  |  |
| Step 1 | | Outcome (1/Death) | | | Death | | | 19 | | 13 | | | 59.4 | |  |
|  |  |  |  |  | Alive | | | 7 | | 598 | | | 98.8 | |  |
|  |  | Overall Percentage | | | | | |  | |  | | | 96.9 | |  |
| a. The cut value is .500 | | | | | | | | | | | | | | |  |
| **Variables in the Equation** | | | | | | | | | | | | | | | |
|  | | | B | S.E. | | Wald | df | | Sig. | | AOR | 95% C.I.for EXP(B) | | | |
|  |  |  |  |  |  |  |  |  |  |  |  | Lower | | Upper | |
| Step 1^a^ | Fever (1) | | 0.172 | 0.720 | | 0.057 | 1 | | 0.811 | | 0.842 | 0.205 | | 3.454 | |
|  | Cough (1) | | 1.220 | 0.885 | | 1.898 | 1 | | 0.168 | | 3.386 | 0.597 | | 19.192 | |
|  | Tiredness (1) | | 2.835 | 0.941 | | 9.078 | 1 | | **0.003** | | **0.059** | **0.009** | | **0.371** | |
|  | Aches and pains (1) | | 2.714 | 1.128 | | 5.786 | 1 | | **0.016** | | **0.066** | **0.007** | | **0.605** | |
|  | Sore throat (1) | | 0.737 | 0.748 | | 0.972 | 1 | | 0 .324 | | 0.478 | 0.110 | | 2.073 | |
|  | Vomiting (1) | | 0.189 | 1.068 | | 0.031 | 1 | | 0.859 | | 1.209 | 0.149 | | 9.804 | |
|  | Females (1) | | 0.856 | 0.415 | | 4.243 | 1 | | **0.030** | | 0.220 | 0.059 | | 0.827 | |
|  | Diabetes Mellitus (1) | | 2.251 | 0.536 | | 17.818 | 1 | | **0.010** | | 9.014 | 1.726 | | 47.067 | |
|  | Other comorbidities (1) | | 1.669 | 0.527 | | 10.042 | 1 | | **0.020** | | 6.860 | 1.309 | | 35.957 | |
|  | Age of 50 years and above (1) | | 1.003 | 0.424 | | 5.588 | 1 | | **0.018** | | 2.725 | 1.187 | | 6.258 | |
|  | Diarrhea (1) | | 1.153 | 1.109 | | 1.080 | 1 | | 0.299 | | 3.167 | 0.360 | | 27.853 | |
|  | Conjunctivitis (1) | | 14.818 | 40192.970 | | 0.000 | 1 | | 1.000 | | 2725229.309 | 0.000 | | . | |
|  | Systolic Blood Pressure | |  |  | |  |  | |  | |  |  | |  | |
|  | Systolic Blood Pressure (1) | | 0.879 | 1.063 | | 0.684 | 1 | | 0.408 | | 0.415 | 0.052 | | 3.334 | |
|  | Systolic Blood Pressure (2) | | 0.236 | 0.874 | | 0.073 | 1 | | 0.787 | | 0.790 | 0.142 | | 4.380 | |
|  | Diastolic Blood Pressure | |  |  | |  |  | |  | |  |  | |  | |
|  | Diastolic Blood Pressure (1) | | 0.127 | 1.127 | | 0.013 | 1 | | 0.910 | | 1.135 | 0.125 | | 10.331 | |
|  | Diastolic Blood Pressure (2) | | 0.758 | 1.128 | | 0.452 | 1 | | 0.501 | | 2.135 | 0.234 | | 19.462 | |
|  | Headache (1) | | 0.127 | 0.618 | | 0.043 | 1 | | 0.836 | | 0.880 | 0.262 | | 2.954 | |
|  | Loss of taste (1) | | 1.694 | 3.308 | | 0.262 | 1 | | 0.609 | | 0.184 | 0.000 | | 120.312 | |
|  | Loss of smell (1) | | 21.360 | 5073.231 | | 0.000 | 1 | | 0.997 | | 1890546855.615 | 0.000 | | . | |
|  | Rash on the skin or discoloration of fingers or toes (1) | | 15.022 | 1294.863 | | 0.000 | 1 | | 0.991 | | 0.000 | 0.000 | | . | |
|  | Difficulty in breathing or shortness of breath  chest pain or pressure  (1) | | 26.271 | 1830.880 | | 0.000 | 1 | | 0.989 | | 0.000 | 0.000 | | . | |
|  | Loss of speech or movement (1) | | 2.008 | 0.812 | | 6.112 | 1 | | **0.013** | | **0.134** | **0.027** | | **.660** | |
|  | Constant | | 31.437 | 1830.880 | | 0.000 | 1 | | 0.986 | | 44972995770729.460 |  | |  | |
| a. Variable(s) entered on step 1: Fever, Cough, Tiredness, Aches and pains, Sore throat, Vomiting, Diarrhoea, Conjunctivitis, Systolic Blood Pressure, Diastolic Blood Pressure, Headache, Loss of taste, Loss of smell, Rash on the skin or discoloration of fingers or toes, Difficulty in breathing or shortness of breath, chest pain or pressure, Loss of speech or movement, Diabetes mellitus, females, other comorbidities, age 50 years and above. | | | | | | | | | | | | | | | |

Step number: 1

Observed Groups and Predicted Probabilities

800 + +

I I

I I

F I I

R 600 + +

E I AI

Q I AI

U I AI

E 400 + A+

N I AI

C I AI

Y I AI

200 + A+

I AI

I AI

I AI

Predicted ---------+---------+---------+---------+---------+---------+---------+---------+---------+----------

Prob: 0 .1 .2 .3 .4 .5 .6 .7 .8 .9 1

Group: DDDDDDDDDDDDDDDDDDDDDDDDDDDDDDDDDDDDDDDDDDDDDDDDDDAAAAAAAAAAAAAAAAAAAAAAAAAAAAAAAAAAAAAAAAAAAAAAAAAA

Predicted Probability is of Membership for Alive

The Cut Value is .50

Symbols: D - Death

A - Alive

Each Symbol Represents 50 Cases.
